# Inhibitory Effects of Quercetin and Its Main Methyl, Sulfate, and Glucuronic Acid Conjugates on Cytochrome P450 Enzymes, and on OATP, BCRP and MRP2 Transporters

**DOI:** 10.3390/nu12082306

**Published:** 2020-07-31

**Authors:** Violetta Mohos, Eszter Fliszár-Nyúl, Orsolya Ungvári, Katalin Kuffa, Paul W. Needs, Paul A. Kroon, Ágnes Telbisz, Csilla Özvegy-Laczka, Miklós Poór

**Affiliations:** 1 Department of Pharmacology, Faculty of Pharmacy, University of Pécs, Szigeti út 12, H-7624 Pécs, Hungary; mohos.violetta@gytk.pte.hu (V.M.); eszter.nyul@aok.pte.hu (E.F.-N.); 2 Lab-on-a-Chip Research Group, János Szentágothai Research Centre, University of Pécs, Ifjúság útja 20, H-7624 Pécs, Hungary; 3 Membrane Protein Research Group, Institute of Enzymology, Research Centre for Natural Sciences, H-1117 Budapest, Hungary; ungvari.orsolya@ttk.mta.hu (O.U.); laczka.csilla@ttk.mta.hu (C.Ö.-L.); 4 Biomembrane Research Group, Institute of Enzymology, Research Centre for Natural Sciences, H-1117 Budapest, Hungary; kuffakatalin@gmail.com (K.K.); telbisz.agnes@ttk.mta.hu (Á.T.); 5 Quadram Institute Bioscience, Norwich Research Park, Norwich NR4 7UQ, UK; paul.needs@quadram.ac.uk (P.W.N.); paul.kroon@quadram.ac.uk (P.A.K.)

**Keywords:** quercetin, quercetin conjugates, cytochrome P450 enzymes, OATP transporters, ABC transporters, pharmacokinetic interaction, food drug interaction

## Abstract

Quercetin is a flavonoid, its glycosides and aglycone are found in significant amounts in several plants and dietary supplements. Because of the high presystemic biotransformation of quercetin, mainly its conjugates appear in circulation. As has been reported in previous studies, quercetin can interact with several proteins of pharmacokinetic importance. However, the interactions of its metabolites with biotransformation enzymes and drug transporters have barely been examined. In this study, the inhibitory effects of quercetin and its most relevant methyl, sulfate, and glucuronide metabolites were tested on cytochrome P450 (CYP) (2C19, 3A4, and 2D6) enzymes as well as on organic anion-transporting polypeptides (OATPs) (OATP1A2, OATP1B1, OATP1B3, and OATP2B1) and ATP (adenosine triphosphate) Binding Cassette (ABC) (BCRP and MRP2) transporters. Quercetin and its metabolites (quercetin-3′-sulfate, quercetin-3-glucuronide, isorhamnetin, and isorhamnetin-3-glucuronide) showed weak inhibitory effects on CYP2C19 and 3A4, while they did not affect CYP2D6 activity. Some of the flavonoids caused weak inhibition of OATP1A2 and MRP2. However, most of the compounds tested proved to be strong inhibitors of OATP1B1, OATP1B3, OATP2B1, and BCRP. Our data demonstrate that not only quercetin but some of its conjugates, can also interact with CYP enzymes and drug transporters. Therefore, high intake of quercetin may interfere with the pharmacokinetics of drugs.

## 1. Introduction

**Figure 1:**
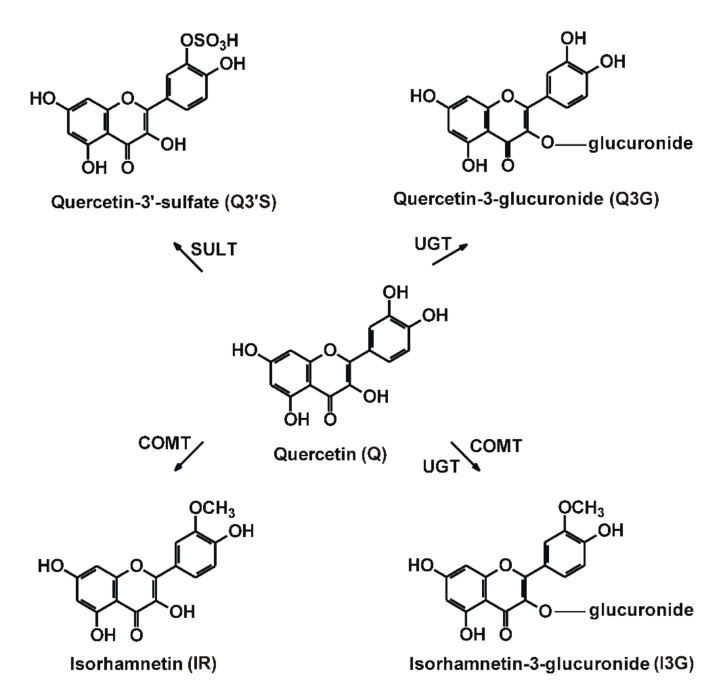
Chemical structures of quercetin (Q), quercetin-3′-sulfate (Q3′S), quercetin-3-glucuronide (Q3G), isorhamnetin (IR), and isorhamnetin-3-glucuronide (I3G). (COMT, catechol-*O*-methyltransferase; SULT, sulfotransferase; UGT, uridine 5′-diphospho-glucuronosyltransferase).

Flavonoids are biologically active natural polyphenols. Quercetin (Q), including its glycosides, is one of the most abundant flavonoids in nature: it is contained in several fruits (e.g., apple, grapes, and berries), vegetables (e.g., onion and tomato), and medicinal plants (e.g., *Ginkgo Biloba* and *Hypericum perforatum*). Because of the cardioprotective and other suspected positive health effects of Q [1], it is widely marketed through the Internet as the active ingredient of dietary supplements [2,3]. These supplements usually contain 200 to 1000 mg aglycone in one single tablet/capsule, and the recommended daily dose is between 250 and 4000 mg [3]. It means even a 100-fold higher intake of Q compared to its estimated average dietary intake (25–50 mg/day) [2,4]. Q has poor oral bioavailability, which shows large variation between individuals [1], partly because of its significant presystemic biotransformation in enterocytes and hepatocytes. As a result of its metabolism by catechol-*O*-methyltransferase (COMT), sulfotransferase (SULT) and uridine 5′-diphospho-glucuronosyltransferase (UGT), its methyl (3′-*O*-methylquercetin or isorhamnetin, IR), sulfate (quercetin-3′-sulfate, Q3′S) and glucuronic acid (quercetin-3-glucuronide, Q3G; isorhamnetin-3-glucuronide, I3G) conjugates are produced (Figure 1) [1]. Human studies suggest that Q3′S, Q3G, and I3G are the dominant circulating metabolites of Q, and the total peak plasma concentration of Q conjugates can achieve 2 μM or even higher levels after the administration of 1000 mg Q daily for one month [5,6,7,8]. Furthermore, based on the study of Kaushik et al. [9], the peak plasma concentrations of total Q were as high as 5 to 10 μM in some human subjects, after the administration of a 500 mg single dose of Q contained by certain oral carrier systems.

Cytochrome P450 (CYP) enzymes have a major role in the metabolism of several endogenous compounds, drugs, and other xenobiotics. CYP3A4 is involved in the biotransformation of more than 50% of the orally administered drugs; furthermore, CYP2C19 and 2D6 are also relevant enzymes in drug metabolism [10]. As has been reported, Q can inhibit CYP enzymes, including CYP1A1, 1B1, 2C9, 2C19, 2D6, and 3A4 [11,12,13]. Furthermore, Q is an inducer of certain CYP enzymes [13]. Some Q metabolites can also interact with CYP. The inhibitory effects of Q3′S, IR, and tamarixetin (4′-*O*-methylquercetin) on CYP2C9 have been reported, while Q3G and I3G did not affect the enzyme [14]. In other studies, the inhibition of CYP1A1, 1A2, 1B1, 2C9, and 3A4 by IR have been also described [15,16,17]. Nevertheless, we did not find data regarding the effects of Q sulfates and glucuronides on CYP2C19, 2D6, and 3A4 enzymes.

Organic anion-transporting polypeptides (OATPs) are solute carrier membrane transporters. They have a major role in the absorption, tissue distribution, and elimination of endogenous molecules (e.g., steroids, bile acids, and bilirubin), drugs (e.g., statins), food components (e.g., Q and naringin), and toxins (e.g., alpha-amanitin and ochratoxin A) [18,19,20,21,22]. OATP1B1, OATP1B3, and OATP2B1 appear in hepatocytes [23]; furthermore, OATP1A2 and OATP2B1 are involved in the uptake of their substrates into enterocytes and/or the central nervous system [19,24,25,26]. The inhibition of OATPs 1A2, 1B1, and 2B1 by Q has been demonstrated in previous reports [27,28]. Furthermore, in vitro studies suggest that Q and IR are substrates of certain OATPs [29,30]. Some Q derivatives (e.g., isorhamnetin-3-glucoside and quercetin-3-*O*-alpha-L-arabinopyranosyl) also proved to be inhibitors of OATP1B1 or OATP2B1 [31,32]. However, the effects of the sulfate/glucuronic acid conjugates of Q and IR on the transport function of multispecific OATPs 1A2, 1B1, 1B3, and 2B1 has not yet been examined.

ATP (adenosine triphosphate) Binding Cassette (ABC) drug transporters, such as P-gp/MDR1, BCRP/ABCG2, and MRP2/ABCC2, are pharmacologically relevant, ATP-driven exporters, which are commonly involved in the urinary/biliary excretion of numerous compounds, including certain metabolites [33,34,35,36,37]. Furthermore, these efflux transporters also have high importance in drug exclusion at placental and blood–brain barriers. Their substrate specificity is partially overlapping; however, P-gp transports mostly hydrophobic compounds, MRP2 transports mainly organic anions and drug conjugates, while BCRP can handle various substrates (many hydrophobic compounds as well as some organic anions and conjugates are its substrates) [34]. As it has been reported earlier, flavonoids are able to inhibit the transport function of ABC transporters, which resulted in the development of strong and specific inhibitors for P-gp or BCRP [38,39,40]. Some flavonoids are transported substrates of BCRP such as flavopiridol or Q [41,42]. MRP2 can also transport some flavonoids, including Q and biochanin A [43,44,45]. In general, the interactions of flavonoids with ABC transporters have been examined in several studies; however, only limited information is available about the sulfate/glucuronide metabolites, due to their poor accessibility and/or technical difficulties regarding their investigations.

In this in vitro study, we aimed to investigate the inhibitory effects of Q and its main conjugates (Q3′S, Q3G, IR, and I3G) on CYP (3A4, 2C19, and 2D6) enzymes as well as on OATP (OATP1A2, OATP1B1, OATP1B3, and OATP2B1) and ABC (BCRP and MRP2) transporters. Our results highlight that the Q metabolites examined can significantly inhibit certain enzymes/transporters tested; therefore, it is reasonable to hypothesize that the simultaneous administration of high dose Q-containing dietary supplements with drugs may result in the development of pharmacokinetic interactions.

## 2. Materials and Methods

### 2.1. Reagents

CypExpress^TM^ 2C19, 2D6 and 3A4 human kits, quercetin (Q), ticlopidine hydrochloride, quinidine, testosterone, 6β-hydroxytestosterone, ketoconazole, fetal bovine serum (FBS), glutamine, penicillin, streptomycin, pyranine (trisodium 8-hydroxypyrene-1,3,6-trisulfonate), bromosulfophthalein, sulforhodamine 101, sodium orthovanadate, probenecid, 5(6)-carboxy-2′,7′-dichlorofluorescein (CDCF), lucifer yellow (LY), and benzbromarone were purchased from Sigma-Aldrich (St. Louis, MO, USA). Nicotinamide adenine dinucleotide phosphate sodium salt (NADP^+^), and glucose-6-phosphate barium salt (G6P) were from Reanal (Budapest, Hungary). S-mephenytoin, 4-hydroxymephenytoin, dextromethorphan, and dextrorphan were obtained from Carbosynth (Berkshire, UK). Isorhamnetin (IR) and Ko143 were purchased from Extrasynthese (Genay Cedex, France) and Tocris Bioscience (Bristol, UK), respectively. Quercetin-3′-sulfate (Q3′S), quercetin-3-glucuronide (Q3G), and isorhamnetin-3-glucuronide (I3G) were synthetized as described previously [46].

### 2.2. CYP Assays

The in vitro inhibitory effects of Q and its conjugates on CYP enzymes were tested, using CypExpress^TM^ Cytochrome P450 human kits (Sigma-Aldrich, St. Louis, MO, USA). Each experiment included solvent controls (DMSO did not exceed 0.6 *v*/*v*%). In CYP2C19, 2D6, and 3A4 assays, Food and Drug Administration (FDA)-recommended substrates (S-mephenytoin, dextromethorphan, and testosterone, respectively) and positive controls (ticlopidine, quinidine, and ketoconazole, respectively) were employed.

Inhibition of CYP2C19 [47], 3A4 [48], and 2D6 [49] by Q and its conjugates was tested based on our previously reported methods, without modifications.

### 2.3. HPLC Analyses

Substrates and the formed metabolites were analyzed by a HPLC system built up from a Waters 510 pump (Milford, MA, USA), a Rheodyne 7125 injector with a 20-µL sample loop (Berkeley, CA, USA), and a Waters 486 UV-detector (Milford, MA, USA). Data were evaluated employing a Waters Millennium Chromatography Manager (Milford, MA, USA).

S-mephenytoin and 4-hydroxymephenytoin (CYP2C19 assay), testosterone and 6β-hydroxytestosterone (CYP3A4 assay), as well as dextromethorphan and dextrorphan (CYP2D6 assay) were quantified using the previously described methods [47,48,49], with minor modifications. Representative chromatograms are demonstrated in Figure S1.

In CYP2C19 assay, the mobile phase contained acetonitrile, methanol (VWR), and sodium phosphate buffer (10 mM, pH 4.55) (17:10:73 *v*/*v*%). Samples were driven through a guard column (Phenomenex Security Guard C8, 4.0 × 3.0 mm) linked to an analytical column (Phenomenex C8 100 × 4.6 mm; 2.6 µm), with a 1 mL/min flow rate, at room temperature. After the isocratic elution, S-mephenytoin and 4-hydroxymephenytoin were detected at 230 nm.

In the CYP3A4 assay, samples were driven through a guard column (Security Guard™ Catridge C18 4.0 × 3.0 mm; Phenomenex, Torrance, CA, USA) linked to an analytical column (Kinetex C18 150 × 4.6 mm; 5 µm; Phenomenex, Torrance, CA, USA), with a 1.2 mL/min flow rate, at room temperature. The mobile phase contained methanol (VWR, Budapest, Hungary), water, and acetic acid (53:46:1 *v*/*v*%). After the isocratic elution, testosterone and 6β-hydroxytestosterone were detected at 240 nm.

In the CYP2D6 assay, samples were driven through a guard column (Security Guard C8, 4.0 × 3.0 mm; Phenomenex, Torrance, CA, USA) linked to an analytical column (Mediterranea Sea8 C8 150 × 4.6 mm; 5 µm; Teknokroma, Barcelona, Spain). The isocratic elution was performed with 1 mL/min flow rate at room temperature, the mobile phase contained acetonitrile and 6.9 mM sodium-acetate buffer (pH 4.0) (31:69 *v*/*v*%). Dextromethorphan and dextrorphan were detected at 280 nm.

### 2.4. OATP Overexpressing Cell Lines and OATP Interaction Tests

A431 cells overexpressing human OATPs, 1A2 (BC042452, HsCD00333163), 1B1 (Gene ID: AB026257), 1B3 (BC141525, HsCD00348132) or 2B1 (BC041095.1, HsCD00378878), or their mock transfected controls, were generated as previously described [50,51]. Cells were cultured in Dulbecco’s Modified Eagle Medium (DMEM; Thermo Fischer Scientific, Waltham, MA, USA) supplemented with 10% fetal bovine serum, 2 mM L-glutamine, 100 units/mL penicillin and 100 µg/mL streptomycin, at 37 °C with 5% CO_2_.

The interaction between flavonoids and OATPs was investigated in an indirect assay [50] employing the fluorescent dye substrates, pyranine and sulforhodamine 101 [51,52]. Briefly, A431 cell overexpressing OATPs, 1A2, 1B1, 1B3 or 2B1, or their mock transfected controls [50,51], were seeded on 96-well plates in a density of 8x10^4^ cells/well in 200 μL DMEM one day prior to the transport measurements. The next day, the medium was removed, then cells were washed three times with 200 μL phosphate-buffered saline (PBS, pH 7.4) and pre-incubated with 50 μL uptake buffer (125 mM NaCl, 4.8 mM KCl, 1.2 mM CaCl_2_, 1.2 mM KH_2_PO_4_, 12 mM MgSO_4_, 25 mM MES (2-(N-morpholino)ethanesulfonic acid), and 5.6 mM glucose, pH 5.5) with or without increasing concentrations of the flavonoids at 37°C. Each test compound was dissolved in DMSO (that did not exceed 0.5 *v*/*v*% in samples), and solvent controls were also applied. The reaction was started by the addition of 50 μL uptake buffer containing pyranine in a final concentration of 10 μM (OATP1B1) or 20 μM (OATP1B3 and OATP2B1), or 0.5 μM sulforhodamine 101 (OATP1A2). Thereafter, cells were incubated at 37°C for 15 min (OATP1B1 and OATP2B1), 10 min (OATP1A2), or 30 min (OATP1B3). The reactions were stopped by removing the supernatants, then the cells were washed three times with ice-cold PBS. Fluorescence (in 200 μL PBS/well) was determined employing an Enspire plate reader (Perkin Elmer, Waltham, MA) ex/em: 403/517 nm (pyranine) or 586/605 nm (sulforhodamine 101). OATP-dependent transport was calculated by extracting fluorescence measured in mock transfected cells. Transport activity was calculated based on the fluorescence signal in the absence (100%) of flavonoids. Experiments were repeated at least in three biological replicates. IC_50_ values were calculated by Hill1 fit, using the Origin Pro8.6 software (GraphPad, La Jolla, CA, USA).

### 2.5. Transport Activity Measurements for MRP2 and BCRP Transporters

Experiments were performed in insect membrane vesicles, as describe previously [53,54,55,56,57]. Human BCRP and MRP2 were expressed in Sf9 insect cells by baculoviruses. At the third day of infection, cells were collected and membrane vesicles were obtained by mechanical disruption and differential centrifugation. To get full activity for BCRP, the cholesterol levels of the vesicles were adjusted to the level of mammalian membranes [57]. Vesicles were stored at -80°C, total protein content of preparations was measured by the Lowry method (used as a reference of the quantity). Membrane vesicles (50 μg protein/sample) were incubated at 37 °C for 10 min (without or with 4 mM of Mg-ATP) in 50 μL volume, in the presence of transporter specific fluorescent substrates (10 μM lucifer yellow (LY) for BCRP and 5 μM 5(6)-Carboxy-2′,7′-dichlorofluorescein (CDCF) for MRP2). Quality of membrane vesicles was confirmed applying known reference inhibitors (benzbromarone and Ko143). ATP dependent uptake of fluorescent substrates was examined in the presence of flavonoids (up to 50–200 μM). Each compound was dissolved in DMSO and solvent controls were applied in all experiments. Under the applied conditions (DMSO did not exceed 2 *v*/*v*% final concentration), it did not affect the measurements. After incubation, samples were rapidly filtered and washed on filter plate (MSFBN6B10, Millipore, Burlington, MA, USA). Accumulated substrates in vesicles were solved back from the filter by 100 μL of 10% sodium dodecyl sulfate and centrifuged into another plate. A 100-μL volume of fluorescence stabilizer was added to the samples (DMSO for LY, and 0.1 M NaOH for CDCF). Fluorescence of samples was measured by a plate reader (Victor X3 Perkin-Elmer, Waltham, MA, USA) at appropriate wavelengths (filters were λ_ex_ = 405 nm and λ_em_ = 535 nm for LY as well as λ_ex_ = 492 nm and λ_em_ = 635 nm for CDCF). ABC related transport was calculated by subtracting passive uptake measured without Mg-ATP (with Mg-AMP the same background was detected) from values measured in the presence of Mg-ATP. Under the applied circumstances, we did not observe considerable quenching effects of flavonoids.

### 2.6. ATPase Activity Assays for BCRP Transporter Interaction

ATPase activity was measured on Sf9 membrane vesicles containing human BCRP prepared as described in 2.5. Appropriate amounts of vesicles (10 μg/50 μL) were used in the assays. Experimental parameters were the same as it has been earlier reported [53,54,55,56,57]. Membrane vesicles were incubated with 3 mM of Mg-ATP for 25 min at 37 °C. Effects of flavonoids were investigated up to 50 μM. Solvent controls were applied in each experiment (DMSO concentration was 2 *v*/*v*% in all samples, which did not modify basal activity). ABC transporter function was determined as vanadate sensitive ATPase activity. Liberated inorganic phosphate was measured by a colorimetric reaction as described [53]. Absorbance of samples was measured after 25 min at 660 nm.

### 2.7. Statistics

Data represent means ± standard error of the mean (SEM) values. Statistical analyses were performed employing one-way ANOVA (*p* < 0.01) with a Tukey’s post-hoc test (IBM SPSS Statistics, Armonk, NY, USA).

## 3. Results

### 3.1. Inhibition of CYP Enzymes by Q and Its Conjugates

**Figure 2:**
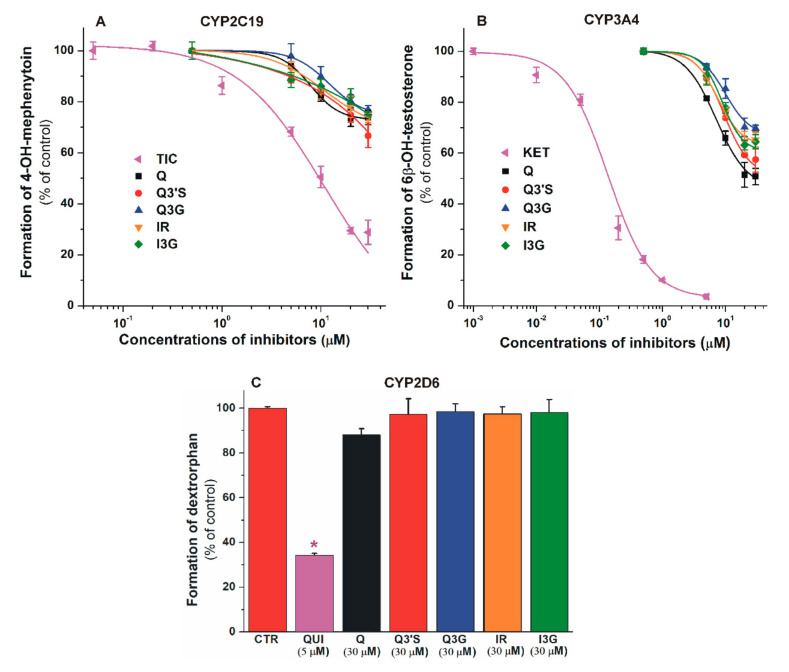
Inhibitory effects of Q, Q3′S, Q3G, IR, I3G, and positive controls on cytochrome P450 (CYP)2C19 (**A**), 3A4 (**B**), and 2D6 ((**C**); * *p* < 0.01) enzymes. Inhibition of CYP2C19-catalyzed S-mephenytoin hydroxylation (positive control: ticlopidine, TIC; IC_50_ = 4.3 μM), CYP3A4-catalyzed testosterone hydroxylation (positive control: ketoconazole, KET; IC_50_ = 0.2 μM), and CYP2D6-catalyzed dextromethorphan *O*-demethylation (positive control: quinidine, QUI; IC_50_ = 0.2 μM) by flavonoids (substrate concentrations: 5 µM in each assay). CYP2C19: statistically significant (p < 0.01) decrease in metabolite formation was induced by 5 μM of TIC, 10 μM of Q, and 20 μM of Q3′S, Q3G, IR, and I3G. CYP3A4: statistically significant (*p* < 0.01) decrease in metabolite formation was induced by 0.05 μM of KET, 5 μM of Q, 20 μM of Q3G, and 10 μM of Q3′S, IR, and I3G (Q, quercetin; Q3′S, quercetin-3′-sulfate; Q3G, quercetin-3-glucuronide; IR, isorhamnetin; I3G, isorhamnetin-3-glucuronide).

The effects of flavonoids (and positive controls) on CYP enzymes are summarized in Figure 2. Each compound tested induced concentration-dependent inhibition of CYP2C19 and 3A4 activity. Q conjugates proved to be similarly strong inhibitors of these enzymes to the parent compound, while their inhibitory effects were considerably weaker compared to the positive controls. Flavonoids showed significant (*p* < 0.01) inhibitory effects on CYP2C19 and 3A4 at 5 to 20 μM concentrations (one- to four-fold concentration vs. the substrates). In the presence of 30 μM flavonoid concentrations, approximately 25% to 35% and 30% to 45% decreases in metabolite formation were observed in CYP2C19 (Figure 2A) and CYP3A4 (Figure 2B) assays, respectively. Thus, under the applied conditions, Q and its metabolites failed to induce 50% or larger inhibitory effects on CYP2C19 and 3A4. The interaction of Q and its conjugates with CYP2D6 was also tested; however, no significant inhibition was noticed, even in the presence of 30 μM flavonoid concentrations (Figure 2C).

### 3.2. Inhibition of OATP Activity by Q and Its Conjugates

**Figure 3:**
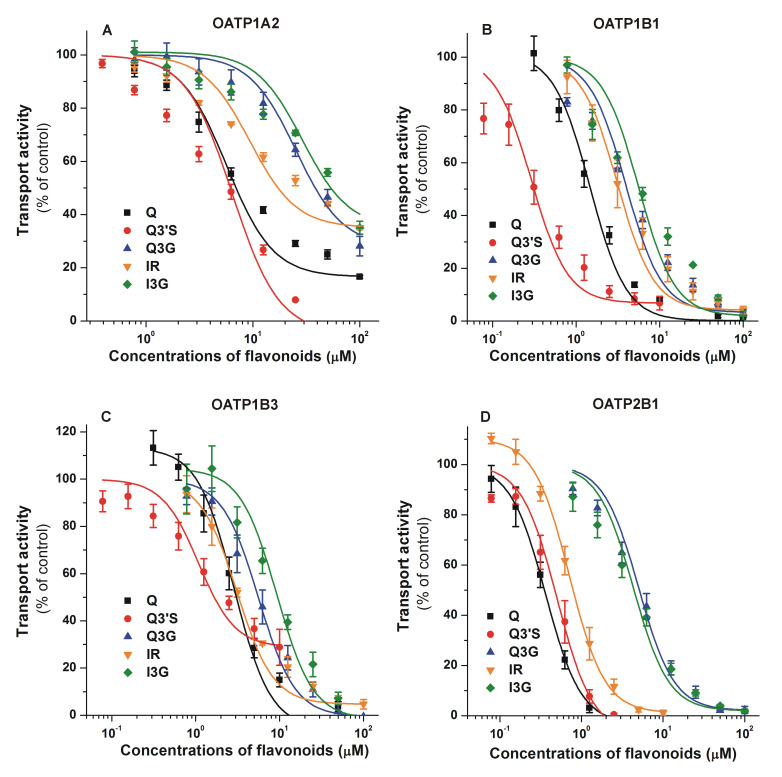
Concentration-dependent inhibitory effects of Q, Q3′S, Q3G, IR, and I3G on the transporter functions of organic anion-transporting polypeptides: OATP1A2 (**A**), OATP1B1 (**B**), OATP1B3 (**C**), and OATP2B1 (**D**). Uptake of pyranine (10 μM for OATP1B1, and 20 μM for OATP1B3 and OATP2B1) or 0.5 μM sulforhodamine 101 (OATP1A2) was measured in A431 cells overexpressing OATPs 1A2, 1B1, 1B3, or 2B1 in the presence of increasing concentrations of the flavonoids for 10 (OATP1A2), 15 (OATP1B1 and OATP2B1), or 30 min (OATP1B3). Fluorescence measured in mock transfected controls was subtracted from the values measured in A431-OATP cells. Fluorescence determined in the absence of the flavonoids was set as 100 %. Mean ± standard error of the mean (SEM) values were obtained from three biological replicates. Regarding Q3′S, we did not use concentrations above 20 μM, since higher levels of Q3′S exhibited fluorescence that interfered with the signal of the test substrate pyranine. The effects of bromosulfophthalein (positive control) on OATPs in the same experimental models have been previously reported [49,51]. OATP1A2: statistically significant (*p* < 0.01) inhibition was caused by 3.1 μM of Q, 0.8 μM of Q3′S, 25 μM of Q3G, 3.1 μM of IR, and 12.5 μM of I3G. OATP1B1: statistically significant (*p* < 0.01) inhibition was induced by 1.3 μM of Q, 0.3 μM of Q3′S, 1.6 μM of Q3G, 3.1 μM of IR, and 1.6 μM of I3G. OATP1B3: statistically significant (*p* < 0.01) inhibition was caused by 2.5 μM of Q, 1.3 μM of Q3′S, 3.1 μM of Q3G, 3.1 μM of IR, and 6.3 μM of I3G. OATP2B1: statistically significant (*p* < 0.01) inhibition was induced by 0.3 μM of Q, 0.3 μM of Q3′S, 3.1 μM of Q3G, 0.6 μM of IR, and 1.6 μM of I3G (Q, quercetin; Q3′S, quercetin-3′-sulfate; Q3G, quercetin-3-glucuronide; IR, isorhamnetin; I3G, isorhamnetin-3-glucuronide).

Inhibitory effects of flavonoids on OATP-mediated dye uptake are summarized in Figure 3. Each flavonoid examined induced a concentration dependent inhibition of OATP1A2 activity: Q3′S and Q showed the strongest impacts (IC_50_ values were 4.9 and 10.1 μM, respectively), while other Q metabolites (Q3G, IR, and I3G) proved to be weak inhibitors of this transporter (Figure 3A). We observed a potent inhibition of OATP1B1 by each flavonoid (Figure 3B), showing low micromolar (Q, Q3G, IR, and I3G) or even nanomolar (Q3′S) IC_50_ values (Table 1). Q conjugates were similarly strong inhibitors of OATP1B3 to the parent compound, each flavonoid caused a 50% decrease in transport activity at low micromolar concentrations (Figure 3C). The IC_50_ values of Q, Q3′S, and IR were in the submicromolar range for OATP2B1 (Table 1). Furthermore, glucuronides (Q3G and I3G) were also strong inhibitors of OATP2B1, with approximately 4 to 5 μM IC_50_ (Figure 3D). Generally, Q3′S was the most potent and glucuronides (Q3G/I3G) were the least effective inhibitors of the OATPs tested (Table 1). Nevertheless, the IC_50_ values of Q3G and I3G were close to 5 μM regarding OATPs 1B1, 1B3, and/or 2B1.

### 3.3. Effects of Q and Its Conjugates on ABC Transporters

**Figure 4:**
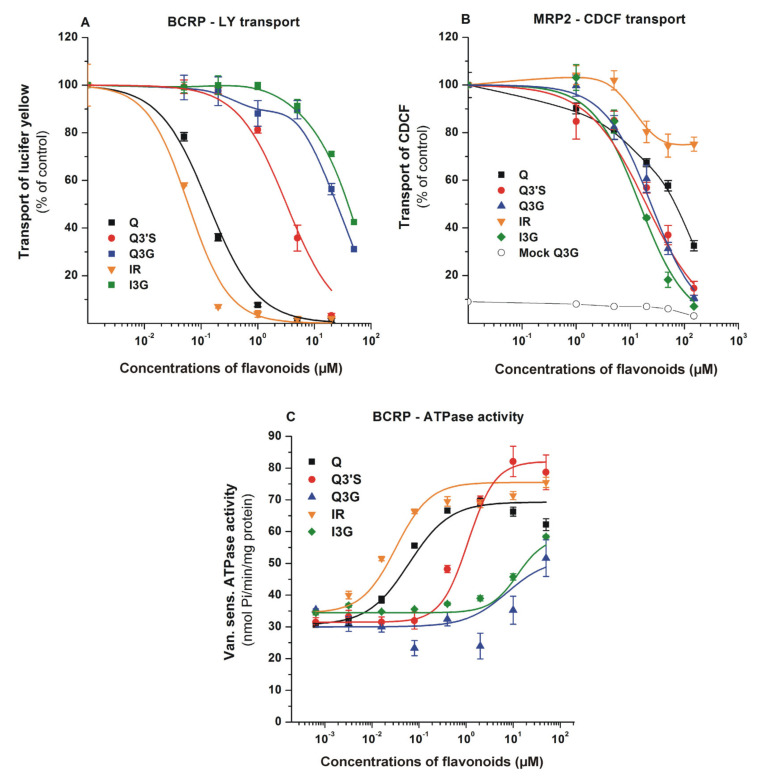
Effects of Q and its metabolites on BCRP and MRP2. BCRP transporter function was measured in human transporters containing inverted insect cell membrane vesicles. Transport activity was characterized by vesicular uptake of a specific fluorescent substrate (lucifer yellow (LY) for ABCG2 and 5(6)-carboxy-2′,7′-dichlorofluorescein (CDCF) for MRP2), where the transport by the ABC transporter is defined as ATP dependent uptake of the substrate into the vesicles. Vanadate sensitive ATPase activity of BCRP was measured by detecting inorganic, deliberated phosphate in a colorimetric reaction. (**A**) Effects of flavonoids on the transport of LY by BCRP. Statistically significant (*p* < 0.01) decrease in transport was induced by 0.05 μM of Q, 0.2 μM of Q3′S, 1.0 μM of Q3G, 0.05 μM of IR, and 20 μM of I3G. (**B**) Effects of flavonoids on the transport of CDCF by MRP2. Flavonoid-induced impacts were tested on mock membranes as well, data in the presence of Q3G are demonstrated (data for other flavonoids were identical). Statistically significant (*p* < 0.01) decrease in transport was caused by 20 μM of each flavonoid. (**C**) Changes in the ATPase activity of BCRP in the presence of flavonoids. Statistically significant (*p* < 0.01) increase in ATPase activity was induced by 0.08 μM of Q, 0.4 μM of Q3′S, 50 μM of Q3G, 0.02 μM of IR, and 10 μM of I3G. Mean ± SEM values were obtained from three biological replicates (Q, quercetin; Q3′S, quercetin-3′-sulfate; Q3G, quercetin-3-glucuronide; IR, isorhamnetin; I3G, isorhamnetin-3-glucuronide).

To test the influence of Q and its conjugates on the transport activity of BCRP and MRP2, vesicular uptake measurements were performed with the fluorescent substrates LY (BCRP) and CDCF (MRP2). The inhibitory effects of flavonoids are demonstrated in Figure 4. The specificity of transports were confirmed employing reference inhibitors, namely benzbromarone for MRP2 and Ko143 for BCRP (data not shown). Since we observed a small background during CDCF transport (MRP2) measurements, mock membrane data for Q3G are also presented (Figure 4B; data for the other compounds were identical, and are not shown). Both BCRP- and MRP2-mediated transport activities were significantly inhibited by the flavonoids tested. Q and IR proved to be potent inhibitors of BCRP, followed by Q3′S which also showed strong inhibition, whereas glucuronides (Q3G and I3G) can be classified as weak inhibitors (Figure 4A). In contrast, MRP2 showed the strongest interactions with glucuronide and sulfate conjugates, while Q and IR were less effective inhibitors (Figure 4B).

Since Q and some of its metabolites were potent inhibitors of BCRP, we also examined BCRP ATPase activity in the presence of flavonoids. BCRP ATPase activity was stimulated by Q and its metabolites (Figure 4C), suggesting that these flavonoids can be substrates and not only inhibitors of the transporter. In good accordance with the transport assay, highest affinity was observed with IR and Q (K_m_ = 0.03 and 0.13 μM, respectively). However, glucuronides showed activation only at high concentrations (100 μM). Based on these observations, both BCRP and MRP2 interact with the Q metabolites investigated.

## 4. Discussion

Based on previously reported positive effects of Q (as an anti-oxidant, anti-inflammatory, and cardioprotective agent), Q-containing dietary supplements are widely marketed through the Internet, and high intake of Q may interfere with drug therapy [2,3,13]. Therefore, there is an urgent need for the deeper understanding of drug interactions regarding Q and its metabolites. In human studies, the pharmacokinetics of certain medications was not affected by Q, while other reports demonstrated that even a single dose of Q can alter the bioavailability of several drugs [2]. As a result of the oral administration of high doses of Q (1500 mg daily for a week) to human subjects, C_max_ and AUC_0-∞_ values of midazolam were declined [58], likely due to the increased CYP3A4-catalyzed elimination of the drug. Furthermore, after seven days treatment with 500 mg Q daily, the oral bioavailability of fexofenadine was significantly increased [59], presumably because of the inhibition of P-gp. However, both single and repeated treatment with Q (1500 mg/day) reduced C_max_ and AUC_0-∞_ values of talinolol in human volunteers [60]. Authors suggested the interactions of Q with both efflux (e.g., P-gp) and uptake (e.g., OATP) transporters; thus, the inhibition of OATP-mediated absorption of talinolol seems to be the dominant mechanism.

The inhibitory effect of Q on CYP enzymes has been reported in several studies, however, some of these results are inconsistent. Previous reports suggest strong [11] or weak [61,62] inhibitory effects of Q on the CYP2C19-catalyzed S-mephenytoin hydroxylation. Furthermore, the significant inhibitory action of Q on CYP3A4-catalyzed midazolam [11] and testosterone [16] hydroxylation has also been demonstrated. In agreement with our current results (Figure 2C), moderate or negligible effects of Q on CYP2D6-mediated bufuralol hydroxylation [11,61] and dextromethorphan *O*-demethylation [62] have been described. In HEK293 cells (overexpressing the respective CYP enzyme), Q inhibited CYP1A1 and 1B1 enzymes, while it did not show relevant inhibition on CYP3A4 and 2D6 [63]. In a few studies, the effects of IR on CYP enzymes have also been examined. Previous in vitro studies regarding CYP3A4 showed controversial results: IR inhibited testosterone hydroxylation (recombinant, human CYP3A4) [16] and 7-benzyloxy-4-trifluoromethylcoumarin *O*-dealkylation (in hepatic microsomes from male and female pigs) [64], while it did not affect 7-benzyloxy-4-trifluoromethylcoumarin *O*-demethylation (tested with both recombinant CYP3A4 and human liver microsomes) [65]. In a recent study, IR did not show an inhibitory effect on the CYP2D6-catalyzed *O*-demethylation of dextromethorphan in vitro [66]. Based on our results, Q and its conjugates are weak inhibitors of CYP2C19 and 3A4, while they did not affect the CYP2D6 enzyme (Figure 2). Therefore, it does not seem likely that Q strongly influences the biotransformation of drugs eliminated via CYP2C19, 3A4, and/or 2D6 enzymes. Nevertheless, a milder inhibition regarding CYP2C19- and/or CYP3A4-mediated elimination of certain drugs cannot be excluded, since not only Q but its metabolites can also exert moderate inhibition on these enzymes. This hypothesis is supported by the report of Bedada and Neerati [67] where the administered Q (500 mg twice daily for 10 days) significantly decreased the CYP2C9-mediated elimination of diclofenac in healthy human subjects, despite the observations in a previous in vitro study that Q and some of its conjugates (Q3′S, IR, and tamarixetin) were only weak inhibitors of the enzyme and glucuronides (Q3G and I3G) did not affect CYP2C9 activity [14]. Previous studies showed the similar or stronger interactions of methyl and sulfate metabolites of Q with xanthine oxidase enzyme [68] and human serum albumin [14,69] compared to the parent compound. Furthermore, Q sulfates and glucuronides showed potent inhibitory effects on organic anion transporters (OAT1 and OAT3) [70].

Transporters of the ABC and OATP family are key participants in the absorption, distribution and elimination of a wide array of chemically diverse compounds. OATP2B1 and OATP1A2 (expressed in enterocytes) may be involved in the absorption of orally consumed/administered flavonoids. Indeed, Duan and colleagues showed transcellular transport of IR, which was inhibited by estrone-3-sulfate (a known substrate of OATPs) [30]. Some other studies suggest the involvement of OATP2B1 and/or other OATPs (1B1/1B3) in the uptake of Q into Caco-2 and HepG2 cells, respectively [43,71]. In addition, Glaeser and colleagues demonstrated direct cellular uptake of radiolabeled Q into HEK-293 cells overexpressing OATPs 1A2, 2A1, 2B1, 1B1, 1B3, 3A1, and 5A1 [29]. Based on these data, OATPs 1B1, 1B3, and 2B1 may contribute to the hepatic uptake then elimination of flavonoids and their conjugates. Furthermore, OATPs 1A2 and 2B1 may also influence the penetration of flavonoids through the blood–brain barrier [25]. A previous study failed to confirm the involvement of OATPs in the uptake of quercetin-7-glucuronide and Q3G into HepG2 cells [72]; however, in this study, the expression of OATPs in HepG2 cells was not confirmed. Wong and colleagues described the OATP4C1-mediated uptake of Q metabolites, including Q3G and Q3′S, into HepG2 cells [70]; nevertheless, this transporter is not a typical OATP in the liver. Therefore, the role of OATPs in the cellular uptake of Q metabolites is not clearly understood yet. Certain food components can influence the function of OATPs and are able to alter the pharmacokinetics of OATP substrate drugs. As a matter of fact, apple, grapefruit, and orange juices are potent inhibitors of intestinal OATP2B1 and OATP1A2, and cause clinically relevant food–drug interactions [19]. In vivo data also show that Q influences the pharmacokinetics of the OATP substrate pravastatin [73]. Herein, we demonstrated that Q and its conjugates can inhibit multispecific OATPs (1B1, 1B3, and 2B1) at submicromolar or low micromolar concentrations (Table 1). Furthermore, Q3′S is a potent inhibitor of OATPs 1B1 and 2B1, because its nanomolar concentrations can strongly decrease transport activities. Similarly, submicromolar IC_50_ values of Q and IR were noticed for OATP2B1. The only exception among OATPs was OATP1A2, which is weakly inhibited by Q, Q3G, IR, and I3G. This latter observation is in good agreement with our recent study, showing that chrysin and its sulfate/glucuronide metabolites are weak inhibitors of OATP1A2, while chrysin conjugates proved to be strong inhibitors of OATPs 1B1, 1B3, and 2B1 [49]. In addition, our data confirm the previous findings obtained with [^3^H]-bromosulfophthalein, [^3^H]-estrone-3-sulfate, and atorvastatin as test substrates [20,73]. On the other hand, these are the first results which demonstrate that sulfate and glucuronic acid conjugates of Q and/or IR can strongly inhibit OATP-mediated transport. Based on previous clinical studies, Q3′S, Q3G, and I3G can achieve high nanomolar or even micromolar concentrations in human circulation [6,7,8], suggesting that these metabolites may be able to induce a relevant inhibition of OATP-mediated uptake of certain compounds from the blood into tissues (e.g., liver and/or brain). Furthermore, some Q conjugates seem to be excreted to the intestinal lumen by ABC transporters from enterocytes and/or through bile [1]; therefore, both the parent compound and its metabolites may appear in the intestinal lumen, and consequently may be able to interfere with the OATP-driven absorption of other compounds. Considering the above-listed data, high intake of Q may affect the OATP-mediated transport (e.g., absorption, hepatic uptake, or blood-brain barrier penetration) of certain drugs. On the other hand, some toxins (e.g., alpha-amanitin and ochratoxin A) are also taken up by OATP transporters into hepatocytes [21,22]; therefore, it is reasonable to hypothesize that circulating Q conjugates may decrease the hepatic uptake of these toxins.

ABC multidrug transporters are responsible for extrusion of drugs and metabolites at the apical surface of cells in important tissue barriers. Studies on the transport of fluorescent or radioactive probe substrates in polarized epithelial cells and transporter overexpressing cell lines showed that P-gp-, BCRP-, and MRP2-related transports are inhibited by Q. In monolayers, selective inhibitors were applied to show the protein specified inhibitions [38,40,74]. Based on blood–brain barrier penetration and monolayer cell experiments, Q seems to be transported by BCRP and MRP2 [40,41,43,74,75]. Although the interactions of certain Q conjugates with BCRP and MRP2 have been reported [27,30,42,44,72,76,77], the characterization of inhibition/transport is far from complete. In the current study, the comparison of IC_50_ values of Q and its metabolites in vesicular transport assay was suitable to reveal the differences in the strength of interactions between ABC transporters and Q derivatives. Both BCRP- and MRP2-mediated transport activities were significantly inhibited by each Q metabolite tested; however, the order of their inhibitory potency were different for these ABC transporters. Q and IR proved to be the most potent inhibitors of BCRP with nanomolar IC_50_ values, and Q3′S also showed strong inhibitory effect at low micromolar concentrations (Table 1). Similarly, the transport-coupled ATPase activity of BCRP was significantly stimulated by low concentrations of Q, IR, and Q3′S (Figure 4C). The same trends and similar concentration dependence were observed in ATPase and transport assays, suggesting that not only Q but its conjugates may be substrates of BCRP. However, direct transport measurements are necessary to confirm this hypothesis. For MRP2, IC_50_ data are higher by an order of magnitude than for BCRP. This difference is generally observed with many other substrates [40,78]. MRP2 is known to expel glucuronide and sulfate conjugates from the cells, whereas hydrophobic compounds are less effectively transported by MRP2. Our current results are in harmony with these previous observations. We found weaker interactions of Q and IR with MRP2 compared to glucuronide and sulfate conjugates. Considering the strong inhibition of BCRP by Q, IR, and Q3′S, it is reasonable to hypothesize that the simultaneous administration of Q may affect the BCRP-mediated transport of some drugs.

In summary, the current study underlines the potent interactions of Q conjugates with pharmacologically important proteins. Considering the above-listed data, Q and/or its metabolites may cause clinically relevant interactions with certain biotransformation enzymes and drug transporters. Therefore, the simultaneous administration of high dose Q-containing dietary supplements with drugs should be carefully considered.

## Figures and Tables

**Table 1:** IC_50_ values of flavonoids for CYP2C19, CYP3A4, OATPs, BCRP, and MRP2.

Proteins	Q	Q3′S	Q3G	IR	I3G
	IC_50_ (μM)	IC_50_ (μM)	IC_50_ (μM)	IC_50_ (μM)	IC_50_ (μM)
CYP2C19	>30.0	>30.0	>30.0	>30.0	>30.0
CYP3A4	>30.0	>30.0	>30.0	>30.0	>30.0
OATP1A2	10.1	4.92	>30.0	>30.0	>30.0
OATP1B1	1.55	0.33	4.05	3.12	5.63
OATP1B3	3.22	2.50	5.71	3.10	9.62
OATP2B1	0.34	0.43	4.85	0.82	4.14
BCRP	0.13	3.20	13.5	0.06	>30
MRP2	>30.0	19.6	24.2	>30.0	14.9

Data were calculated based on measurements demonstrated in Figures 2–4. Q, quercetin; Q3′S, quercetin-3′-sulfate; Q3G, quercetin-3-glucuronide; IR, isorhamnetin; I3G, isorhamnetin-3-glucuronide.

## Supplementary Materials

The following are available online at https://www.mdpi.com/2072-6643/12/8/2306/s1, Figure S1: Representative chromatograms of S-mephenytoin and 4-hydroxymephenytoin (CYP2C19 assay), dextromethorphan and dextrorphan (CYP2D6 assay) as well as testosterone and 6β-hydroxytestosterone (CYP3A4 assay) (each 2.5 μM).
