# Supplementary material for: Inhibitory Effects of Quercetin and Its Main Methyl, Sulfate, and Glucuronic Acid Conjugates on Cytochrome P450 Enzymes, and on OATP, BCRP and MRP2 Transporters
Source: Nutrients. 2020 Jul 31;12(8):2306. doi: 10.3390/nu12082306 (PMC7468908; doi:10.3390/nu12082306)
Supplement: Supplementary file 1 [file nutrients-12-02306-s001.pdf]

nutrients

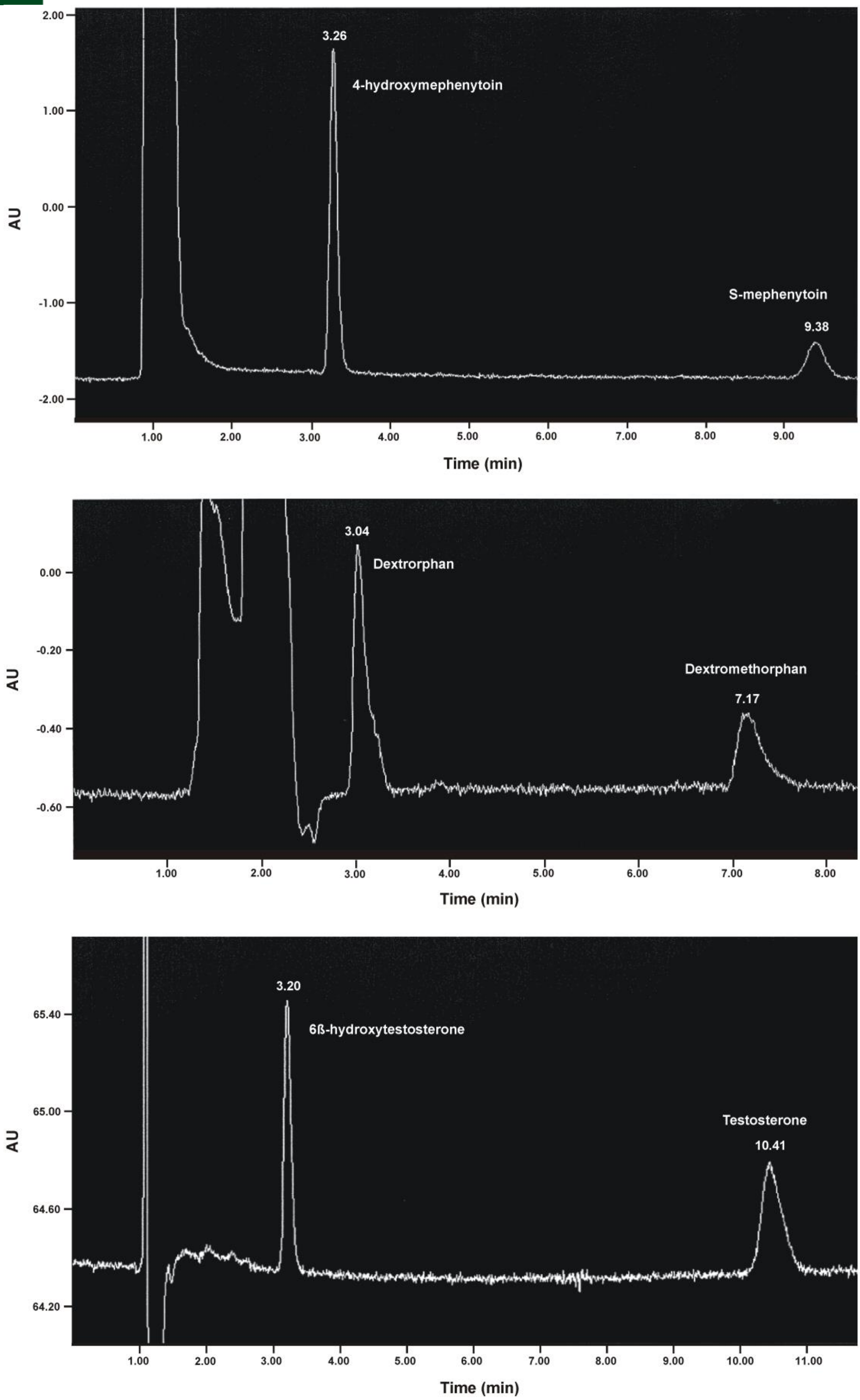
2.00
1.00
0.00
-1.00
-2.00
AU
3.26
4-hydroxymephenytoin
S-mephenytoin
9.38
1.00
2.00
3.00
4.00
5.00
6.00
7.00
8.00
9.00
Time (min)
0.00
-0.20
-0.40
-0.60
AU
3.04
Dextrorphan
Dextromethorphan
7.17
1.00
2.00
3.00
4.00
5.00
6.00
7.00
8.00
Time (min)
65.40
65.00
64.60
64.20
AU
3.20
6ß-hydroxytestosterone
Testosterone
10.41
1.00
2.00
3.00
4.00
5.00
6.00
7.00
8.00
9.00
10.00
11.00
Time (min)

Figure S1. Representative chromatograms of S-mephenytoin and 4-hydroxymephenytoin (top, CYP2C19 assay), dextromethorphan and dextrorphan (middle, CYP2D6 assay) as well as testosterone and 6β-hydroxytestosterone (bottom, CYP3A4 assay) (each 2.5 μM).
